# Association between central non-dipping pattern and platelet morphology in adults with type 1 diabetes without cardiovascular disease: a cross-sectional study

**DOI:** 10.1038/s41598-021-94414-y

**Published:** 2021-07-29

**Authors:** Michal Kulecki, Dariusz Naskret, Mikolaj Kaminski, Dominika Kasprzak, Pawel Lachowski, Daria Klause, Maria Kozlowska, Justyna Flotynska, Aleksandra Uruska, Dorota Zozulinska-Ziolkiewicz

**Affiliations:** Department of Internal Medicine and Diabetology, Poznan University of Medical Sciences, ul. Mickiewicza 2, 60-834 Poznań, Poland

**Keywords:** Cardiology, Diabetes

## Abstract

The non-dipping pattern is nighttime systolic blood pressure (SBP) fall of less than 10%. Several studies showed that the non-dipping pattern, increased mean platelet volume (MPV), and platelet distribution width (PDW) are associated with elevated cardiovascular risk. Hypertensives with the non-dipping pattern have higher MPV than the dippers but this relationship was never investigated among people with type 1 diabetes mellitus (T1DM). This study aimed to investigate the association between the central dipping pattern and platelet morphology in T1DM subjects. We measured the central and brachial blood pressure with a validated non-invasive brachial oscillometric device—Arteriograph 24—during twenty-four-hour analysis in T1DM subjects without diagnosed hypertension. The group was divided based on the central dipping pattern for the dippers and the non-dippers. From a total of 62 subjects (32 males) aged 30.1 (25.7–37) years with T1DM duration 15.0 (9.0–20) years, 36 were non-dippers. The non-dipper group had significantly higher MPV (MPV (10.8 [10.3–11.5] vs 10.4 [10.0–10.7] fl; *p* = 0.041) and PDW (13.2 [11.7–14.9] vs 12.3 [11.7–12.8] fl; *p* = 0.029) than dipper group. Multivariable logistic regression revealed that MPV (OR 3.74; 95% CI 1.48–9.45; *p* = 0.005) and PDW (OR 1.91; 95% CI 1.22–3.00; *p* = 0.005) were positively associated with central non-dipping pattern adjusting for age, sex, smoking status, daily insulin intake, and height. MPV and PDW are positively associated with the central non-dipping pattern among people with T1DM.

## Introduction

Type 1 diabetes mellitus (T1DM) is a chronic metabolic illness, where insulin treatment is obligatory^1^. People with T1DM have a higher risk of developing cardiovascular disease than those from the general population^2^. Women with T1DM die 12.5 years earlier than women without diabetes. In the case of men, the difference is 11.6. years^3^. However, the pathophysiology of elevated cardiovascular risk in this population is still uncertain.

The circadian rhythm of blood pressure (BP) is useful to predict cardiovascular events^4,5^. The non-dipping pattern is defined as nighttime systolic blood pressure (SBP) fall of less than 10%^6^. It is usually described based on brachial systolic blood pressure but central compared with brachial SBP is more strongly associated with preclinical organ damage^7^. Non-dippers have more cardiovascular events and higher cardiovascular mortality than dippers, even if they are normotensives^4^.

Until now mean platelet volume (MPV) and platelet distribution width (PDW) could not be used for diagnosis or prognosis in any disease because of poor standardization and wide variability^8^. Mean platelet volume is dependent on numerous physiological and pathological factors^9^ However, many studies showed a positive relation between MPV, the presence of cardiovascular disease, and future prognosis. Based on metanalysis (40 studies, 12 285 individuals) subjects with coronary artery disease have higher MPV than healthy controls^10^. Higher values of MPV and PDW were significantly related to higher risks of cardiovascular disease^11^. MPV could predict long-term outcomes and cardiovascular events after the percutaneous coronary intervention^12^. The morphology and function of platelets in diabetes (including T1DM) are disturbed^13,14^. Rodriguez et al. showed that MPV was positively related to the presence of T2DM independently of age, sex, hypertension, BMI, and use of anti-platelet medicaments^15^. Children with T1DM have significantly higher MPV and PDW than healthy controls^16^.

Previous studies revealed the association between elevated MPV, PDW, and non-dipping pattern among people with hypertension^17–22^. However, a large study by Pusuroglu et al.^23^ did not confirm that thus this relationship remains controversial. There is no data according to people without diagnosed hypertension. This association was never investigated in the T1DM population with a non-dipping pattern based on central SBP.

## Methods

### Data collection

The study aimed to investigate the association between central dipping pattern and platelet morphology in subjects with T1DM. The data comes from the Poznan Atherosclerosis in Adult Patients with long-term Type 1 Diabetes Mellitus Study (PARADISE T1DM Study), which was conducted according to the decision of the Ethics Committee at Poznan University of Medical Sciences (approval No. 67/19). The study complies with the Declaration of Helsinki. In all cases, we obtained written informed consent before inclusion in the study.

The participants of the study were managed by the Department of Internal Medicine and Diabetology. We recruited them consecutively from February 2019 to March 2020. We included people aged between 18 and 45 years with T1DM confirmed in the past by positive antibodies, at least 5-year diabetes duration, and no less than 70% successful blood pressure measurements. The exclusion criteria were: cardiovascular disease, hypertension (or using antihypertensives), malignancy, chronic kidney disease (stages 2–5), sleep apnea, thrombocythemia, or thrombocytopenia**.** Each subject was asked to fill two forms, including diabetes duration, diabetic complications, drugs taken, coexisting diseases, and lifestyle.

Every participant underwent anamnesis and a standard physical examination. Specific investigations were performed to detect diabetic complications: retinopathy, neuropathy, and diabetic kidney disease. Besides, we repeated the brachial blood pressure measurement. The measurement was performed three times by an upper-arm manual sphygmomanometer on both arms after 10 min of rest in the sitting position, at the level of the participant’s heart. Basic anthropometric parameters (weight, height, waist, and hip circumferences) were measured. We calculated body mass index (BMI) and waist-to-hip ratio (WHR) using the following equations: BMI = weight [kg]/ squared height [m^2^]; WHR = waist circumference [cm]/ hip circumference [cm].

All blood samples were taken from participants in the morning (between 7 and 8 AM), after an 8–12-h overnight fast. They were drawn into standardized tubes with dipotassium ethylenedinitrotetraacetic acid (EDTA). The laboratory measured MPV, PDW, and all other lab parameters within 120 min after blood collection. Then the blood samples were stored at room temperature (18–25 °C) Complete blood count was carried out on analyzer Sysmex XN1000 (Sysmex Corporation, Japan). To eliminate pre-analytical and analytical errors, the technicians in the laboratory were not informed if the patient is the dipper or the non-dipper. Each blood sample was collected, handled, and processed in the same way. MPV reflects the average size of platelets. PDW was counted from a platelet histogram at the level of 20% of the distribution peak. The same blood counters were used to mark the morphology of red blood cells and white blood cells.

We also obtained the following laboratory results: lipid profile, thyroid-stimulating hormone, creatinine, transaminases. Low-density lipoprotein cholesterol (LDL-C) level was estimated by the Friedewald formula^24^. Glycated hemoglobin (HbA1c) was evaluated with a turbidimetric inhibition immunoassay (Cobas 6000, Roche Diagnostics). Furthermore, the urine albumin to urine creatinine ratio (ACR) was calculated. Glucose Disposal Rate (eGDR) formula was chosen to calculate insulin resistance^25^: eGDR [mg/kg/min] = 24.31 − (12.22 × WHR) – (3.29 × hypertension) – (0.57 × HbA1c) where: WHR—waist to hip ratio, hypertension—if present count as 1, if not count as 0, HbA1c—glycated hemoglobin [%].

Skin autofluorescence (which reflects advanced glycation end products accumulation and long-term diabetic control^26^) was measured using a non-invasive device (AGE-Reader, DiagnOptics BV, Groningen, The Netherlands). This method was validated by Meerwaldt et al.^27^.

24-h BP measurements were taken using Arteriograph 24 (TensioMed Ltd., Budapest, Hungary). We marked: aortic systolic blood pressure (SBP Ao), brachial systolic blood pressure (SBP Br), brachial diastolic blood pressure (DBP Br), and pulse. The operating principle of Arteriograph 24 is detecting and processing oscillations on the arm cuff by a special high-fidelity sensor during a complete occlusion of the brachial artery^28^. Arteriograph 24 was validated using invasive and non-invasive methods of AS assessment^28,29^.

An appropriate cuff was chosen based on arm circumference. We measured the distance from the jugular notch to the pubic symphysis in the supine position to estimate the length of the aorta (required to calculate Pulse wave velocity). Pulse wave velocity is the gold standard of assessment of arterial stiffness. More rigid arterial walls result in faster pulse wave velocity^30^.

We used TensioWin software to program Arteriograph 24. Measurements were taken every 30 min daily and every 1 h nightly for 24 h during the hospitalization in the clinic. Participants were instructed to start manual measurement in case of failed automatic measurement. After 24 h, the device was removed, and the results were transferred to our database.

Each participant was asked to fill another questionnaire concerning hour by hour activity during 24 h of measurement, including physical activity, number of cigarettes, number of (caffeine-containing) coffee cups, body position in the moment of measurement, glycemia (at least 5 measurements per day), and insulin dosages. We also marked the time of sleeping. Sleeping time was double-checked—using data from Arteriograph 24 and self-reported information from the questionnaire filled by the participant.

Subjects with a nighttime SBP fall of less than 10% were included in the group of non-dippers. Entities with the nighttime SBP fall of at least 10% were involved in the group of dippers.

### Data analysis

R-programming language (version 3.6.1.; Vienna, R Project) was used for statistical analysis. The data is presented either as number and percentage (categorical) or where appropriate as a median and interquartile range (numerical). We used the Chi-square and the Mann–Whitney U tests to compare the dippers and the non-dippers based on central and brachial SBP. *p* value was set as two-sided.

We performed a logistic regression analysis. The dependent variable was the presence of a central or brachial non-dipping pattern. All variables independent of BP were chosen for univariate regression analysis. Univariate regression results with *p* < 0.1 and other known non-dipping pattern risk factors were included in multivariable regression analysis^31^. PDW was included in another model because it was highly positively associated with MPV. We also excluded potassium because of the collinearity with the height.

## Results

### Study group

**Figure 1:**
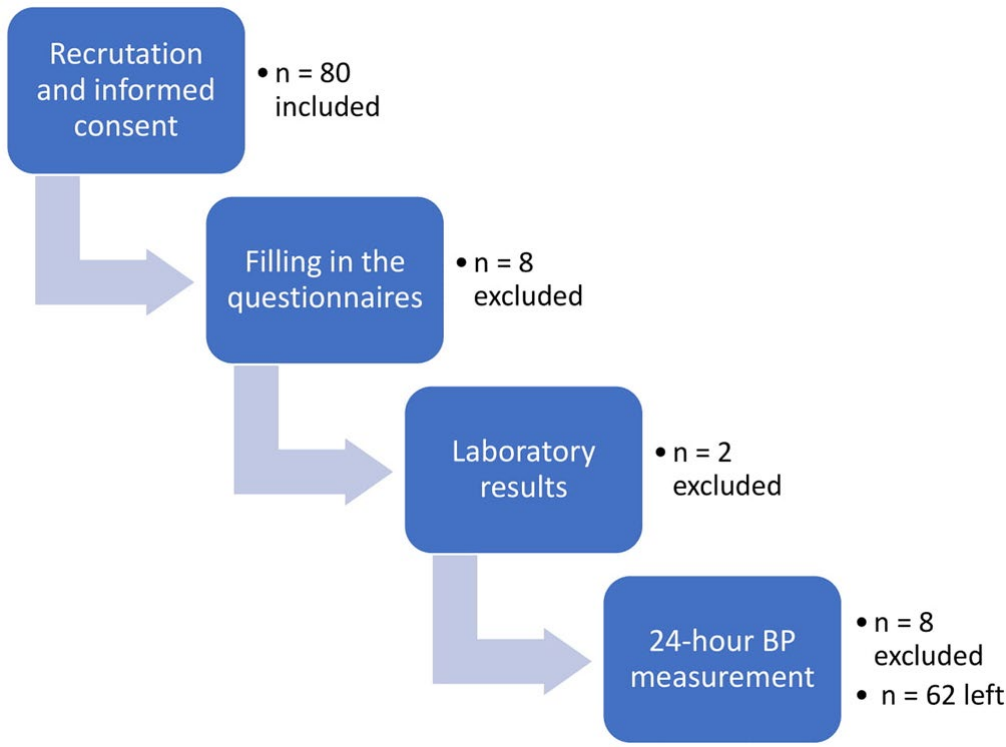
Flow diagram of the study and order of the procedures. *BP* blood pressure. All figures made by authors of the manuscript. GraphPad Prism 9 (https://www.graphpad.com/) was used to create forest plots.

80 participants were recruited. However, we finally included 62 subjects in the final analysis*.* The scheme of the study is presented in Fig. 1. The participants were aged 30.1 (25.7–37.0) years*,* 32 (51.6%) of them were males*.* The median T1DM duration *was* 15.0 (9.0–20.0) years. 36 (58.1%) were non-dippers based on SBP Ao; 25 (40.3%) were non-dippers based on SBP Br.

### Central non-dipping pattern

**Figure 2:**
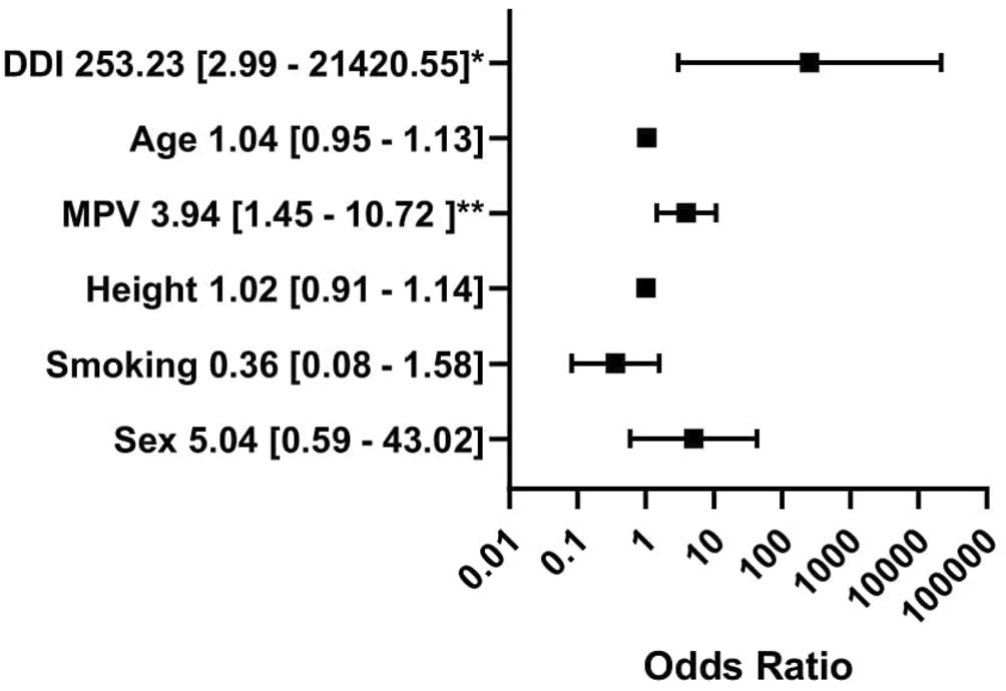
Multivariable logistic regression analysis with mean platelet volume. The dependent variable: the presence of central non-dipping pattern (coded one). The outcomes presented as a dependent variable (odds ratio, 95% confidence interval). *Significant at the 0.05 level; **significant at the 0.01 level. *MPV* mean platelet volume, *DDI* daily insulin intake. All figures made by authors of the manuscript. GraphPad Prism 9 (https://www.graphpad.com/) was used to create forest plots.

**Figure 3:**
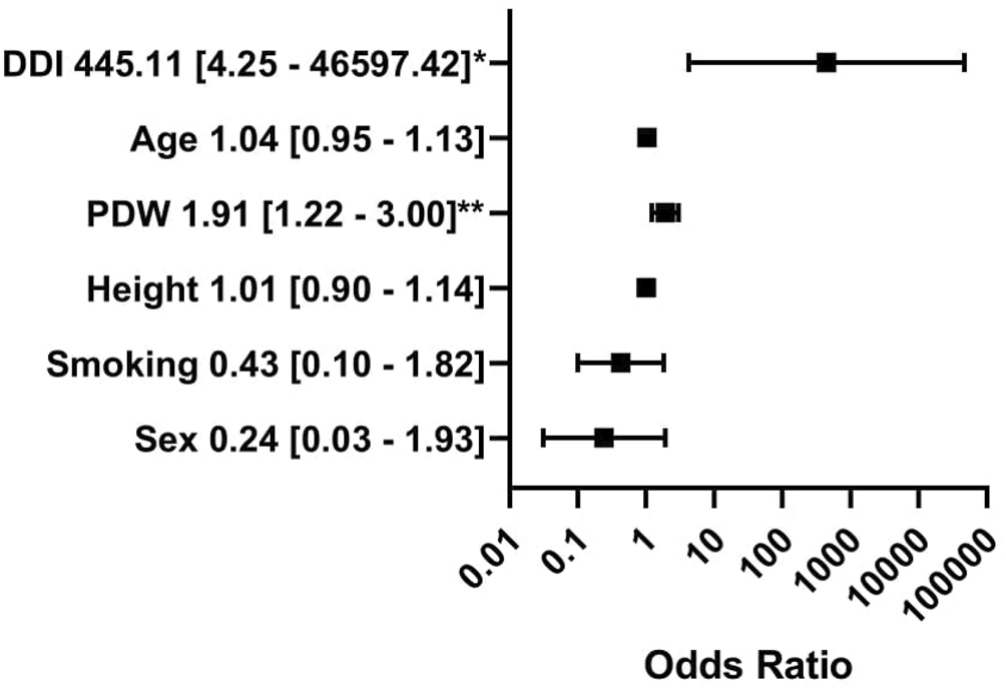
Multivariable logistic regression analysis with platelet distribution width. The dependent variable: the presence of central non-dipping pattern (coded one). The outcomes presented as a dependent variable (odds ratio, 95% confidence interval). *Significant at the 0.05 level. **Significant at the 0.01 level. *PDW* platelet distribution width, *DDI* daily insulin intake. All figures made by authors of the manuscript. GraphPad Prism 9 (https://www.graphpad.com/) was used to create forest plots.

The groups (non-dippers vs dippers based on central SBP) did not differ significantly in general characteristics, diabetes complications, methods of treatment, and activity during the day of measurement. The non-dipper group had significantly higher MPV (10.8 [10.3–11.5] vs 10.4 [10.0–10.7] fl; *p* = 0.041) and PDW (13.2 [11.7–14.9] vs 12.3 [11.7–12.8] fl; *p* = 0.029) than the dipper group. The non-dippers consumed more alcohol than the dippers(1.5 [0.0–4.0] vs 0.8 [0.0–1.0] units/week; *p* = 0.015). The groups did not differ significantly in hemodynamic parameters like pulse wave velocity, aortic systolic blood pressure, brachial systolic and diastolic blood pressure. The differences in other morphology parameters like the number of platelets, red and white blood cells, hemoglobin, hematocrit, platelet volume/ platelet count ratio, as well as lipids, were also insignificant. The inverse associations between platelet count and MPV (Rs =  − 0.17; *p* = 0.18) as well as PDW (Rs =  − 0.15; *p* = 0.24) were not significant. Results are presented in Table 1. In univariate regression analysis (dependent variable—presence of SBP Ao non-dipping pattern) MPV, PDW and daily insulin intake were significant (*p* < 0.05). Sex, height, potassium, and alanine aminotransferase had a trend nearing significance (*p* < 0.1). Potassium concentration was positively associated with height (Rs = 0.34; *p* = 0.007), therefore potassium was excluded from the final model of multivariable logistic regression. PDW was included in another model because it was highly positively associated with MPV (Rs = 0.94; *p* < 0.001). Multivariable logistic regression revealed that MPV (OR 3.94; 95% CI 1.45–10.72; *p* = 0.007) and daily insulin intake (OR 253.23; 95% CI 2.99–21,420.55; *p* = 0.01) were positively associated with non-dipping pattern based on SBP Ao adjusting for age, sex, smoking status and height (Fig. 2). The model with PDW showed the positive relationship between PDW (OR 1.91; 95% CI 1.22–3.00; *p* = 0.005), daily insulin intake (OR 445.11; 95% CI 4.25–46,597.42; *p* = 0.01) and central non-dipping pattern adjusting for age, sex, smoking status and height (Fig. 3).

**Table 1:** Comparison of groups with and without the central non-dipping pattern according to the Arteriograph 24 results.

	All participants n = 62 (100%)	SBP Ao dippers n = 26 (41.9%)	SBP Ao non–dippers n = 36 (58.1%)	*p* value
**General characteristics**				
Males n (%)	32 (51.6%)	10 (38.5%)	22 (61.1%)	0.08
Age [years]	30.1 (25.7–37.0)	29.6 (22.3–39.4)	32.8 (27.5–36.8)	0.59
Diabetes duration [years]	15.0 (9.0–20.0)	16.0 (9.0–22.0)	15.0 (8.5–18.5)	0.43
BMI [kg/m^2^]	24.8 (22.7–28.2)	25.9 (23.2–28.7)	24.3 (22.1–28.2)	0.31
WHR	0.9 (0.8–0.9)	0.9 (0.8–0.9)	0.9 (0.8–0.9)	0.92
Family history for CVD n (%)	17 (27.4%)	5 (19.2%)	12 (33.3%)	0.22
Systolic blood pressure [mmHg]	123.0 (120.0–131.0)	123.0 (116.0–131.0)	123.0 (120.0–132.0)	0.84
Diastolic blood pressure [mmHg]	80.0 (76.0–86.0)	80.0 (76.0–85.0)	81.0 (77.0–86.5)	0.43
**Lifestyle**				
Current smoker n (%)	15 (24.2%)	6 (23.1%)	9 (25%)	0.86
Packyears	0.0 (0.0–68.0)	0.0 (0.0–40.0)	2.3 (0.0–95.0)	0.28
Sport activity [hours/week]	2.4 (0.0–6.0)	2.0 (1.0–7.0)	3.0 (0.0–6.0)	0.86
Alcohol intake [units/week]	**1.0 (0.0–2.0)**	**0.8 (0.0–1.0)**	**1.5 (0.0–4.0)**	**0.015**
Shift work n (%)	22 (35.5%)	10.0 (38.5%)	12.0 (33.3%)	0.68
Sleeping [hours/day]	7.0 (6.0–8.0)	7.0 (6.0–8.0)	7.0 (6.0–8.0)	0.90
Physical work [hours/day]	3.0 (0.0–8.0)	2.0 (0.0–8.0)	3.0 (0.0–8.0)	0.51
**Complications**				
At least one diabetic complication n (%)	34 (54.8%)	13 (50%)	21 (58.3%)	0.52
Diabetic retinopathy n (%)	12 (19.4%)	4 (15.4%)	8 (22.2%)	0.50
Diabetic nephropathy n (%)	2 (3.2%)	1 (3.8%)	1 (2.8%)	0.81
Diabetic neuropathy n (%)	28 (45.2%)	13 (50%)	15 (41.7%)	0.52
**Treatment**				
Insulin pump n (%)	25 (40.3%)	10 (38.5%)	15 (41.7%)	0.95
Daily insulin intake [insulin units/day/kg]	0.6 (0.5–0.7)	0.6 (0.5–0.6)	0.6 (0.5–0.8)	0.09
Metformin n (%)	6 (9.7%)	2 (7.7%)	4 (11.1%)	0.65
**Lipid profile**				
Triglycerides [mmol/l]	1.0 (0.7–1.3)	1.0 (0.7–1.4)	0.9 (0.6–1.3)	0.61
Total cholesterol [mmol/l]	4.6 (4.2–5.1)	4.6 (4.2–5.1)	4.4 (4.2–5.2)	0.95
LDL–C [[mmol/l]	2.4 (2.0–2.8)	2.4 (2.0–2.8)	2.4 (2.0–3.1)	0.89
HDL–C [mmol/l]	1.7 (1.5–1.9)	1.7 (1.4–1.8)	1.8 (1.5–2.0)	0.33
Triglycerides / HDL–C ratio	1.4 (0.8–2.1)	1.3 (0.8–2.1)	1.4 (0.9–2.1)	0.53
**Laboratory results**				
Red blood cells [T/l]	4.8 (4.6–5.2)	4.8 (4.6–5.0)	4.8 (4.6–5.3)	0.33
Hemoglobin [g/l]	14.3 (13.3–15.2)	14.3 (12.8–15.1)	14.2 (13.5–15.2)	0.52
Hematocrit [%]	42.2 (39.2–44.1)	42.0 (38.5–44.1)	42.7 (39.5–44.8)	0.41
Mean red blood cells volume [fl]	85.8 (82.9–88.2)	85.8 (83.3–87.3)	85.5 (82.9–87.9)	0.96
White Blood Cells [G/l]	6.3 (5.5–7.3)	6.0 (5.4–7.2)	6.4 (5.5–7.3)	0.49
Platelets [G/l]	262.0 (245.0–308.0)	269.0 (245.0–315.0)	260.5 (246.0–305.0)	0.68
Mean platelet volume [fl]	**10.6 (10.1–11.2)**	**10.4 (10.0–10.7)**	**10.8 (10.3–11.5)**	**0.041**
Platelet distribution width [%]	**12.5 (11.7–14.2)**	**12.3 (11.7–12.8)**	**13.2 (11.7–14.9)**	**0.029**
HbA1c [%]	8.2 (7.0–9.1)	7.9 (7.2–8.8)	8.5 (7.0–9.7)	0.24
ALT [UI/l]	16.0 (12.0–20.0)	15.0 (11.0–17.0)	16.5 (12.5–24.5)	0.24
AST [UI/l]	17.0 (15.0–21.0)	17.0 (15.0–21.0)	17.0 (15.0–20.5)	0.70
Creatinine [µmol/L]	73.4 (67.2–85.7)	73.8 (66.3–84.9)	72.9 (68.1–86.6)	0.73
ACR [mg/g]	3.6 (3.0–7.0)	4.0 (3.0–9.0)	3.5 (3.0–6.0)	0.38
Estimated glucose disposal rate [mg/kg/min]	9.2 (8.0–10.3)	9.1 (8.1–10.4)	9.3 (7.8–10.2)	0.59
Skin autofluorescence [IU]	2.1 (1.8–2.4)	2.0 (1.8–2.2)	2.1 (1.9–2.5)	0.35
MPV/PLT ratio	0.041 (0.034–0.044)	0.039 (0.031–0.043)	**0.041 (0.036–0.045)**	0.24
**Activity during the day of measurement**				
Time of physical activity [min]	20.0 (0.0–40.0)	20.0 (0.0–50.0)	20.0 (0.0–40.0)	0.93
Number of cigarettes [n]	0.0 (0.0–2.0)	0.0 (0.0–0.0)	0.0 (0.0–4.0)	0.52
Number of coffee cups [n]	2.0 (1.0–3.0)	2.0 (1.0–2.0)	2.0 (1.0–3.0)	0.48
Mean glycemia [mmol/l]	7.5 (6.7–8.8)	7.7 (6.7–8.9)	7.2 (6.8–8.0)	0.50
SD of glycemia [mmol/l]	2.5 (2.0–3.0	2.6 (2.0–3.5)	2.5 (2.0–2.9)	0.25
Insulin/day (u)	38.1 (30.5–46.0)	36.0 (30.5–44.5)	39.0 (31.5–48.0)	0.27
Carbohydrates/day (g)	15.3 (11.8–18.5)	14.0 (12.0–17.5)	16.0 (11.5–19.0)	0.56
**Hemodynamic parameters**				
Aortic systolic blood pressure [mmHg]	118.9 (113.9–125.5)	119.1 (113.9–125.5)	118.7 (113.9–126.0)	0.75
Brachial systolic blood pressure [mmHg]	130.3 (123.9–138.2)	129.2 (121.5–138.2)	130.6 (124.4–138.2)	0.64
Brachial diastolic blood pressure [mmHg]	75.5 (71.1–79.4)	75.2 (68.4–77.6)	75.6 (73.0–79.5)	0.19
Pulse wave velocity [ms/s]	7.7 (7.1–8.5)	7.8 (7.1–8.4)	7.7 (7.1–8.5)	0.88

Data presented as median (IQR)/n(%). Bold values denote statistical significance at the p < 0.05 level.

*ACR* albumin to creatinine ratio, *ALT* alanine transaminase, *Ao* aortic, *AST* aspartate transaminase, *BMI* body mass index, *HbA1c* glycated hemoglobin, *HDL-C* high-density lipoprotein, *LDL-C* low-density lipoprotein, *SBP* systolic blood pressure, *WHR* waist-to-hip ratio.

### Brachial non-dipping pattern

**Figure 4:**
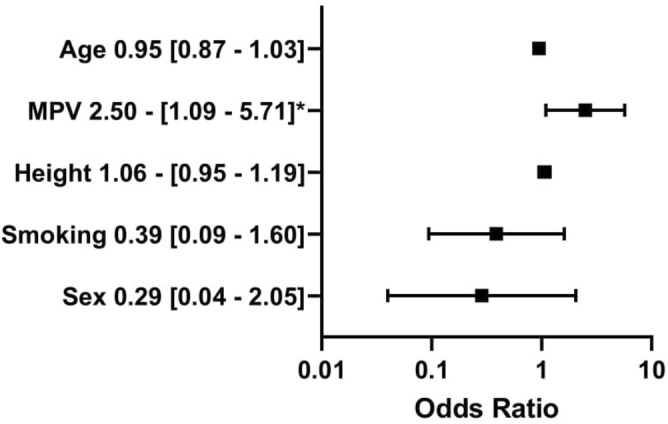
Multivariable logistic regression analysis with mean platelet volume. The dependent variable: the presence of brachial non-dipping pattern (coded one). The outcomes presented as a dependent variable (odds ratio, 95% confidence interval). *. significant at the 0.05 level; **significant at the 0.01 level; *MPV* mean platelet volume, *DDI* daily insulin intake. All figures made by authors of the manuscript. GraphPad Prism 9 (https://www.graphpad.com/) was used to create forest plots.

**Figure 5:**
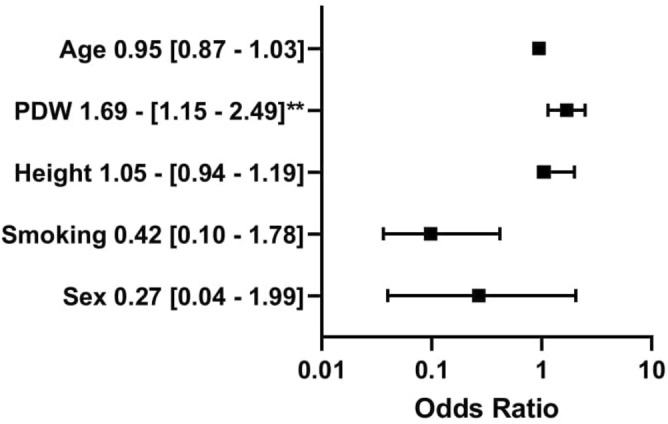
Multivariable logistic regression analysis with platelet distribution width. The dependent variable: the presence of brachial non-dipping pattern (coded one). The outcomes presented as a dependent variable (odds ratio, 95% confidence interval). *Significant at the 0.05 level; **significant at the 0.01 level; *PDW* platelet distribution width, *DDI* daily insulin intake. All figures made by authors of the manuscript. GraphPad Prism 9 (https://www.graphpad.com/) was used to create forest plots.

The comparison of non-dippers and dippers based on brachial SBP revealed the difference in PDW—which was higher in the non-dipper group—(13.9 [12.1–15.1] vs 12.3 [11.7–13.4] fl; *p* = 0.020) but not in MPV (10.7 [10.3–11.4] vs 10.4 [10.0–10.9] *p* = 0.11). In the SBP Br non-dipper group were significantly more males than in the dipper group (17 [68.0%] vs 15 [40.5%] *p* = 0.034) (Table 2). The groups did not differ significantly in aortic and brachial blood pressure and pulse wave velocity. The differences in other morphology parameters like the number of platelets, red and white blood cells, hemoglobin, hematocrit, platelet volume/ platelet count ratio, as well as lipids, were also insignificant. In univariate regression analysis (dependent variable—presence of SBP Br non-dipping pattern) PDW, sex, potassium, and height were significant (*p* < 0.05). Multivariable logistic regression revealed that only MPV (OR 2.50; 95% CI 1.09–5.71; *p* = 0.008) and PDW in another model (OR 1.69; 95% CI 1.15–2.49; *p* = 0.008) were positively associated with non-dipping pattern based on SBP Br adjusting for age, sex, smoking status, and height (Figs. 4 and 5).

**Table 2:** Comparison of groups with and without the brachial non-dipping pattern according to the Arteriograph 24 results.

	All participants n = 62 (100%)	SBP Br dippers n = 37 (57.7%)	SBP Br non–dippers n = 25 (40.3%)	*p* value
**General characteristics**				
Males n (%)	**32 (51.6%)**	**15 (40.5%)**	**17 (68.0%)**	**0.034**
Age [years]	30.1 (25.7–37.0)	30.0 (26.3–37.3)	30.3 (24.0–36.4	0.38
Diabetes duration [years]	15.0 (9.0–20.0)	16.0 (9.0–20.0)	15.0 (8.0–18.0)	0.38
BMI [kg/m^2^]	24.8 (22.7–28.2)	25.8 (23.2–28.4)	23.8 (21.5–28.0)	0.28
WHR	0.9 (0.8–0.9)	0.8 (0.8–0.9)	0.9 (0.8–0.9)	0.71
Family history for CVD n (%)	17 (27.4%)	10 (27.0%)	7 (28.0%)	0.93
Systolic blood pressure [mmHg]	123.0 (120.0–131.0)	121.0 (115.0–130.0)	123.0 (120.0–136.0)	0.22
Diastolic blood pressure [mmHg]	80.0 (76.0–86.0)	82.0 (76.0–85.0)	80.0 (77.0–87.0)	0.48
**Lifestyle**				
Current smoker n (%)	15 (24.2%)	10 (27.0%)	5 (20.0%)	0.53
Packyears	0.0 (0.0–68.0)	0.0 (0.0–80.0)	0.0 (0.0–60.0)	0.93
Sport activity [hours/week]	2.4 (0.0–6.0)	2.0 (0.0–6.0)	3.3 (0.0–6.0)	0.76
Alcohol intake [units/week]	1.0 (0.0–2.0)	1.0 (0.0–1.0)	2.0 (0.0–3.0)	0.050
Shift work n (%)	22 (35.5%)	13 (35.1%)	9 (36.0%)	0.94
Sleeping [hours/day]	7.0 (6.0–8.0)	7.0 (6.0–8.0)	7.0 (6.0–8.0)	0.80
Physical work [hours/day]	3.0 (0.0–8.0)	3.3 (0.0–8.0)	3.0 (0.0–8.0)	0.87
**Complications**				
At least one diabetic complication n (%)	34 (54.8%)	21 (56.8%)	13 (52.0%)	0.71
Diabetic retinopathy n (%)	12 (19.4%)	5 (13.5%)	7 (28.0%)	0.16
Diabetic nephropathy n (%)	2 (3.2%)	1 (2.7%)	1 (4.0%)	0.78
Diabetic neuropathy n (%)	28 (45.2%)	19 (51.4%)	9 (36.0%)	0.23
**Treatment**				
Insulin pump n (%)	25 (40.3%)	14 (37.8%)	11 (44.0%)	0.75
Daily insulin intake [insulin units/day/kg]	0.6 (0.5–0.7)	0.6 (0.5–0.6)	0.6 (0.5–0.7)	0.72
Metformin n (%)	6 (9.7%)	3 (8.1%)	3 (12%)	0.61
**Lipid profile**				
Triglycerides [mmol/l]	1.0 (0.7–1.3)	0.9 (0.6–1.3	1.0 (0.8–1.4)	0.39
Total cholesterol [mmol/l]	4.6 (4.2–5.1)	4.6 (4.2–5.1)	4.6 (4.2–5.0)	0.70
LDL–C [[mmol/l]	2.4 (2.0–2.8)	2.5 (2.2–2.9)	2.2 (1.9–2.8)	0.25
HDL–C [mmol/l]	1.7 (1.5–1.9)	1.7 (1.5–1.9)	1.8 (1.4–1.9)	0.76
Triglycerides / HDL–C ratio	1.4 (0.8–2.1)	1.3 (0.8–2.1)	1.4 (0.9–2.1)	0.65
**Laboratory results**				
Red Blood Cells [T/l]	4.8 (4.6–5.2)	4.8 (4.6–5.0)	4.8 (4.6–5.5)	0.30
Hemoglobin [g/l]	14.3 (13.3–15.2)	14.2 (13.2–15.1)	14.3 (13.5–15.6)	0.32
Hematocrit [%]	42.2 (39.2–44.1)	42.1 (38.7–44.0)	42.5 (39.8–45.5)	0.26
Mean red blood cells volume [fl]	85.8 (82.9–88.2)	85.8 (83.3–87.3)	85.8 (82.9–87.5)	1.00
White blood cells [G/l]	6.3 (5.5–7.3)	6.3 (5.5–7.4)	6.3 (5.5–6.9)	0.95
Platelets [G/l]	262.0 (245.0–308.0)	263.0 (246.0–314.0)	259.0 (230.0–302.0)	0.45
Mean platelet volume [fl]	10.6 (10.1–11.2)	10.4 (10.0–10.9)	10.7 (10.3–11.4)	0.11
Platelet distribution width [%]	**12.5 (11.7–14.2)**	**12.3 (11.7–13.4)**	**13.9 (12.1–15.1)**	**0.020**
HbA1c [%]	8.2 (7.0–9.1)	7.9 (7.2–8.9)	8.5 (7.0–9.9)	0.24
ALT [UI/l]	16.0 (12.0–20.0)	15.0 (13.0–19.0)	16.0 (11.0–24.0)	0.67
AST [UI/l]	17.0 (15.0–21.0)	17.0 (15.0–20.0)	16.0 (15.0–21.0)	0.75
Creatinine [µmol/L]	73.4 (67.2–85.7)	71.6 (67.2–80.4)	75.1 (70.7–86.6)	0.25
ACR [mg/g]	3.6 (3.0–7.0)	3.2 (3.0–7.0)	5.0 (3.0–7.0)	0.93
Estimated glucose disposal rate [mg/kg/min]	9.2 (8.0–10.3)	9.4 (8.1–10.3)	8.8 (8.0–10.1)	0.38
Skin Autofluorescence [IU]	2.1 (1.8–2.4)	2.1 (1.9–2.4)	2.0 (1.7–2.3)	0.50
MPV/PLT ratio	0.041 (0.034–0.044)	0.039 (0.033–0.043)	**0.041 (0.036–0.045)**	0.24
**Activity during the day of measurement**				
Time of physical activity [min]	20.0 (0.0–40.0)	30.0 (10.0–40.0)	10.0 (0.0–30.0)	0.09
Number of cigarettes [n]	0.0 (0.0–2.0)	0.0 (0.0–3.0)	0.0 (0.0–1.0)	0.89
Number of coffee cups [n]	2.0 (1.0–3.0)	2.0 (1.0–3.0)	2.0 (1.0–3.0)	0.50
Mean glycemia [mmol/l]	7.5 (6.7–8.8)	7.4 (6.8–8.0)	7.6 (6.5–9.0)	0.82
SD of glycemia [mmol/l]	2.5 (2.0–3.0)	2.5 (1.9–2.9)	2.5 (2.2–3.1)	0.26
Insulin/day (u)	38.1 (30.5–46.0)	37.9 (30.5–44.9)	39.0 (31.7–46.0)	0.79
Carbohydrates/day (g)	15.3 (11.8–18.5)	15.0 (12.8–17.8)	16.8 (11.0–20.0)	0.82
**Hemodynamic parameters**				
Aortic systolic blood pressure [mmHg]	118.7 (113.9–126.0)	118.9 (113.9–125.5)	119.0 (113.9–125.4)	0.97
Brachial systolic blood pressure [mmHg]	130.6 (124.4–138.2)	130.6 (123.9–135.8)	129.4 (124.0–138.3)	0.79
Brachial diastolic blood pressure [mmHg]	75.6 (73.0–79.5)	75.7 (69.5–78.4)	75.5 (73.3–79.6)	0.47
Pulse wave velocity [ms/s]	7.7 (7.1–8.5)	7.7 (7.1–8.3)	7.8 (6.8–8.7)	0.95

Data presented as median (IQR) / n(%). Bold values denote statistical significance at the p < 0.05 level.

*ACR* albumin to creatinine ratio, *ALT* alanine transaminase, *AST* aspartate transaminase, *BMI* body mass index, *Br* brachial, *HbA1c* glycated hemoglobin, *HDL-C* high-density lipoprotein, *LDL-C* low-density lipoprotein, *SBP* systolic blood pressure, *WHR* waist-to-hip ratio.

### Hypertension in 24-h measurement

All of our participants had no diagnosed hypertension but 61.3% of them had elevated BP values in 24-h measurement. Therefore, we performed an additional analysis for participants without hypertension in 24-h measurement. SBP Ao non dippers had significantly higher MPV (11.0 [10.5–11.6] vs 10.5 [10.0–10.7] fl; *p* = 0.026) and PDW (14.0 [12.5–15.1] vs 12.0 [11.7–12.6] fl; *p* = 0.035) (Table 3).

**Table 3:** Participants without hypertension in 24-h BP measurement. Comparison of the dippers and the non-dippers.

	All participants n = 24 (100%)	SBP Ao dippers n = 13 (54.2%)	SBP Ao non–dippers n = 11 (45.8%)	*p* value
**Laboratory results**				
Mean Platelet Volume [fl]	**10.7 (10.4–11.3)**	**10.5 (10.0–10.7)**	**11.0 (10.5–11.6)**	**0.026**
Platelet distribution Width [%]	**12.5 (11.9–14.4)**	**12.0 (11.7–12.6)**	**14.0 (12.5–15.1)**	**0.035**

Data presented as median (IQR)/n (%). Bold values denote statistical significance at the p < 0.05 level.

## Discussion

To our knowledge, this is the first study investigating the relationship between platelet morphology and the central non-dipping pattern in the T1DM population. We found that MPV and PDW were significantly higher in the central non-dippers compared to the dippers among people with T1DM. MPV and PDW were positively associated with the presence of the central non-dipping pattern adjusting for age, sex, smoking status, and height.

There are different methods to assess platelet reactivity, but many of them are complicated and high-priced^32^. The gold standard of platelet reactivity measurement is turbidimetric platelet aggregation, but there are many other techniques like flow cytometry, shear-stress mediated systems, imaging-based techniques^33,34^. Some studies suggest that the alternative could be MPV, as an indirect method of platelet activity measurement. Higher MPV correlates to a higher number of dense bodies per platelet, LDH activity, speed of ADP-induced aggregation, serotonin uptake, and release^35^. However, the relationship between MPV, PDW, and platelet aggregation was not proved in healthy volunteers^36^. De Luca et al. demonstrated that MPV value does not accompany platelet reactivity in the population of 1016 diabetic patients^37^. This study had several limitations. The diagnosis of diabetes was based only on medical history and fasting plasma glucose. There were no exclusion criteria and no differentiation for the type of diabetes. Furthermore, the results showed the positive association between MPV and U46619-stimulated P-selectin increase with the trend nearing significance (r = 0.24, *p* = 0.11). Therefore, we need more studies to verify the association between platelet activation and MPV, especially in the T1DM population. The limitation of MPV is that many other factors could influence its value: genetic variants, race, ethnicity, age, sex, and lifestyle^9^. PDW and MPV are not simply surrogates of more specific platelet reactivity tests. They reflect different aspects of platelet biology, not only the reactivity of platelets but also their age, generation, and turnover rates^33^.

Hypertension may partially derive from a hypercoagulable state, one component of which may be increased platelet activity. Hypertensives have higher MPV and a higher quantity of P-selectin and beta-thromboglobulin than normotensives. These proteins take part in platelet activation^38^. Gang et al. in a prospective study with a 9-years follow-up proved that elevated MPV allows predicting the development of hypertension independently of other risk factors like age, gender, and SBP^39^. Prehypertension is also associated with higher MPV^40^.

A Chinese prospective study in a large group of 31,751 people revealed that higher values of MPV were associated with a higher risk of stroke, cardiovascular disease, and coronary heart disease after 6 years of follow-up. Higher PDW was related to a higher risk of cardiovascular disease and coronary heart disease^11^. Higher MPV is also associated with a higher risk of restenosis after percutaneous transluminal coronary angioplasty^12^.

Both the non-dipping pattern and increased MPV are associated with elevated cardiovascular risk^5,41^. However, the pathophysiology of the relationship between the non-dipping pattern and MPV is unknown. The non-dipping pattern is related to higher nighttime SBP and the same higher mean diurnal SBP. Increased BP may lead to endothelium damage which results in elevated shear stress, an imbalance between free radicals and antioxidants, platelet activation, and aggregation^42^. Another possible pathomechanism is the impairment of the autonomic nervous system—low parasympathetic and high sympathetic activity. Catecholamines may stimulate alpha-2 adrenoreceptors which induce platelet aggregation^43^. This theory also suggests that adrenaline causes the release of the large activated platelets sequestered in the spleen^44^.

It is known that the presence of diabetes is associated with disturbed platelet biology. MPV was positively associated with the presence of T2DM in both sexes, especially in males^15^. Elevated MPV was related to insulin treatment, lowered MPV was related to sulphonylureas and metformin. In another sample, the researchers observed no relationship between platelet aggregation traits (ADP, collagen, epinephrine) and the risk of diabetes development after 18.1 years of follow-up. King et al. also found no significant association between T2DM and platelet reactivity tests^45^. It may suggest that MPV is a better predictor of T2DM than platelet reactivity tests. Shimodaira et al. proved that MPV was positively related to fasting plasma glucose among both individuals with prediabetes and healthy people with normal glucose levels^46^ In children with T1DM MPV was related to aortic intima-media thickness which is an early indicator of subclinical atherosclerosis^47^. Abdel-Moneim et al. compared three groups of children: with established T1DM (duration: 0.66 ± 0.4 years), with newly diagnosed T1DM (duration: 0.66 ± 0.4 years), and healthy controls. MPV and PDW were significantly higher in children with T1DM than those in the control group^48^. Platelet number was lower in children with established T1DM than in children with the newly diagnosed disease and controls. MPV was related to HbA1C among people with T1DM as opposed to T2DM subjects^13^. Children with HbA1C above 7.5% had significantly higher MPV and PDW than children with HbA1C below 7.5%. MPV was positively correlated with T1DM duration^14^.

The relationship between platelet morphology and the dipping pattern among T1DM subjects is uninvestigated. Our study is the first one and revealed the need for further research. Nevertheless, there are some works focused on the dipping pattern in this population. Theilade et al. investigated for the first time the central dipping among subjects with T1DM^49^. They proved that nocturnal central and brachial SBP fall decreased with diabetes duration and albuminuria. There was an association between HbA1C and the non-dipping pattern^50^. However, Mamta et al. observed no relationship between the brachial non-dipping pattern and glucose variability in subjects with T1DM^51^. Another study showed that nephropathy (but not other diabetic complications) was positively associated with the brachial non-dipping pattern^52^. Spallone et al. suggested the role of autonomic neuropathy in the development of nephropathy and the non-dipping pattern in the T1DM subjects, but further studies did not confirm that relationship^52,53^. In our study, we did not observe such associations. The groups did not differ in long-term diabetic control (HbA1C, skin autofluorescence), presence of diabetic complications, and insulin resistance (eGDR). However, the central non-dipping pattern was positively related to daily insulin intake. The whole study group was relatively young and had a low level of albumin to creatine ratio. It suggests that the non-dipping pattern may develop independently of glycemic control.

The prevalence of the non-dipping pattern among people with T1DM is uninvestigated because few studies have been published on this topic. It was noted by Jaiswal et al.^51^. In their study, the prevalence of the non-dipping pattern in 41 T1DM subjects was only 10%. A previous study by Stella et al. showed that 27.8% of 61 T1DM entities were the non-dippers^52^. The prevalence was 40.3% for the brachial non-dipping pattern and 58.1% for the central non-dipping pattern in our study.

In the literature, several works describe the association between MPV and the non-dipping pattern, but all of them are based only on brachial BP. MPV is thought to be higher in the non-dippers than the dippers among people with hypertension treated at least 6 months^17^. Inanc et al. demonstrated that the non-dippers have higher MPV than the dippers and normotensives^18^. Other studies confirmed the positive relationship between MPV and the non-dipping patterns among hypertensives^19,20,54^. A similar finding was obtained among hypertensive children, people with uncontrolled hypertension, and prehypertensive non-smokers^21,55^ Meric et al. observed that the mean platelet volume/ platelet count (MPV/PC) ratio was significantly higher in the non-dippers than the dippers and it could be used to predict non-dipping pattern with 95 sensitivity and 95 specificity^22^. In our study MPV was higher in central the non-dippers than the dippers. There were no significant differences in MPV based on the brachial dipping pattern. However, MPV was associated with a brachial non-dipping pattern in the multivariable regression model. We found no association between dipping pattern and MPV/PC ratio.

Previously described studies were performed in relatively small populations. On the contrary, Pusuroglu et al. found no difference in MPV between the hypertensive dippers and the non-dippers in the group of 840 participants^23^. However, MPV was significantly higher in hypertensives compared to normotensives. The non-dipping pattern was diagnosed only in hypertensives, so there was no information on how many subjects without hypertension were the non-dippers. We included only subjects with T1DM and without diagnosed hypertension. In our study group, there was no difference in MPV between the dippers and the non-dippers based on peripheral BP, but MPV was significantly higher in the central BP non-dippers than the dippers. Further studies are needed to confirm if MPV is more strongly associated with central than brachial dipping.

Cicek et al. found that hypertensives with the non-dipping pattern have higher pulse wave velocity than those with the dipping pattern^56^. Increased pulse wave velocity means higher arterial stiffness which is a strong cardiovascular risk factor. However, our results showed no relationship between the non-dipping pattern and pulse wave velocity. It could be a result of a smaller number of participants in our study or a different population (with type 1 diabetes and without hypertension). The relationship between the non-dipping pattern and pulse wave velocity is poorly documented yet and it needs further investigation.

In our study, we excluded people with alcoholism. However, the non-dippers consumed significantly more standard units of alcohol per week than the dippers. Even small amounts of alcohol may be associated with the non-dipping pattern but because of the cross-sectional character of our study, we cannot find any cause and effect relationships.

We showed for the first time the relationship between the non-dipping pattern and MPV among people with T1DM. Moreover, this is the first study investigating not only the brachial but central non-dipping pattern and its association with platelet morphology. Several studies proved that the brachial non-dippers have higher MPV than the dippers. We also showed that PDW (which was not investigated earlier) is higher in people without appropriate nocturnal fall in BP.

Those results bring practical implications because people with the non-dipping pattern have elevated cardiovascular risk. Thanks to 24-h BP measurement, PDW, and MPV, it is possible to identify them. We know that in the non-dippers impaired platelet biology should be suspected. These people should be watchfully observed. Non-pharmacological treatment could be useful because 20-weeks lifestyle modification (decreased sodium intake, intensified physical activity, reduction of alcohol consumption, and DASH diet) may reduce MPV in prehypertensive subjects^57^. Aspirin and other antiplatelet drugs are not recommended in primary prevention among T1DM subjects with low or moderate cardiovascular risk^58^. According to the ASCEND study, aspirin brings more side effects than benefits, in primary cardiovascular prevention among people with diabetes^59^. However, this data is based mostly on subjects with type 2 diabetes mellitus and the topic remains controversial. People with diabetes with the non-dipping pattern and increased MPV may have benefits from using antiplatelet drugs. Further studies are needed to establish proper treatment for T1DM subjects with a non-dipping pattern.

Our study has some limitations. We did not measure cardiac autonomic neuropathy, which could influence on dipping pattern. However, studies did not confirm the relationship between non-dipping pattern and autonomic neuropathy^52^. We did not perform more complicated, direct methods of platelet activity assessment. Another limitation of our study is the lack of standardization of MPV and PDW assessment. MPV measurements are cheap and widely available but susceptible to many factors. Firstly, the contact of the platelets with the standard anticoagulant—ethylenediaminetetraacetic acid (EDTA) may cause an increase in diameter^60,61^ Secondly, the MPV measurements are highly variable across analyzers^61,62^. Therefore, the results should be treated with caution cause MPV measurements may be not consistent across different studies. Our results were obtained using the same method and the same analyzer. The groups did not differ significantly in age and gender. The technicians in the laboratory were unconscious if the participant was the dipper or the non-dipper. The time between blood sampling and analysis was similar (within 120 min) but it was not accurately measured^63^. Each participant had only 1 day of 24-h BP measurement. Our results are limited because of the relatively small sample size, modest *p* values, and no account of multiple test correction in analysis.

## Conclusions

Type 1 diabetes subjects with the central non-dipping pattern have higher values of MPV and PDW than the dippers. MPV and PDW are positively associated with the central non-dipping pattern adjusting for age, sex, smoking status, and height.

## Supplementary Information

Supplementary Information.

## Data Availability

The datasets analyzed during the current study are available from the corresponding author on reasonable request.
