# Supplementary material for: Association between central non-dipping pattern and platelet morphology in adults with type 1 diabetes without cardiovascular disease: a cross-sectional study
Source: Sci Rep. 2021 Jul 29;11:15416. doi: 10.1038/s41598-021-94414-y (PMC8322100; doi:10.1038/s41598-021-94414-y)
Supplement: Supplementary file 1 — Supplementary Information. [file 41598_2021_94414_MOESM1_ESM.docx]

**Title: Association between central non-dipping pattern and platelet morphology in adults with type 1 diabetes without cardiovascular disease: a cross-sectional study.**

Authors: Michal Kulecki&*^1^. Dariusz Naskret&^1^. Mikolaj Kaminski^1^. Dominika Kasprzak^1^. Pawel Lachowski^1^. Daria Klause^1^. Maria Kozlowska^1^. Justyna Flotynska^1.^ Aleksandra Uruska#^1^. Dorota Zozulinska-Ziolkiewicz#^1^

# – equal senior author

& -equal first author

* -corresponding author

Supplementary Table S1.

Univariate logistic regression analysis. The dependent variable: the presence of non-dipping pattern. Dependent variable: SBP Ao non-dipping pattern

| Variable | Odds ratio | Confidence interval OR -95% | Confidence interval OR 95% | p-value |
| --- | --- | --- | --- | --- |
| Males n (%) | 2.51 | 0.89 | 7.09 | 0.08 |
| Age [years] | 1.02 | 0.95 | 1.09 | 0.61 |
| Diabetes duration [years] | 0.96 | 0.90 | 1.04 | 0.32 |
| BMI [kg/m^2^] | 1.01 | 0.96 | 1.06 | 0.68 |
| Waist circumference [m] | 0.99 | 0.94 | 1.04 | 0.62 |
| WHR | 1.37 | 0.00 | 1203.04 | 0.93 |
| At least one diabetic complication n (%) | 1.40 | 0.51 | 3.86 | 0.52 |
| Microangiopathy n (%) | 2.19 | 0.52 | 9.22 | 0.28 |
| Diabetic retinopathy n (%) | 1.57 | 0.42 | 5.90 | 0.50 |
| Diabetic nephropathy n (%) | 0.71 | 0.04 | 11.97 | 0.82 |
| Diabetic neuropathy n (%) | 0.71 | 0.26 | 1.97 | 0.52 |
| Insulin pump n (%) | 0.99 | 0.33 | 2.96 | 0.99 |
| **Daily insulin intake [insulin units/day/kg]** | **43.75** | **1.22** | **1569.28** | **0.04** |
| Metformin n (%) | 1.50 | 0.25 | 8.88 | 0.65 |
| Current smoker n (%) | 1.11 | 0.34 | 3.63 | 0.86 |
| Packyears | 1.00 | 1.00 | 1.01 | 0.42 |
| Alcohol intake [units/week] | 1.08 | 0.94 | 1.23 | 0.27 |
| Shift work n (%) | 0.80 | 0.28 | 2.29 | 0.68 |
| Sleeping [hours/day] | 0.96 | 0.61 | 1.52 | 0.87 |
| Physical work [hours/day] | 1.05 | 0.92 | 1.19 | 0.50 |
| Sport activity [hours/week] | 0.99 | 0.90 | 1.10 | 0.87 |
| Red Blood Cells [T/l] | 1.84 | 0.59 | 5.78 | 0.29 |
| Hemoglobin [g/l] | 1.20 | 0.84 | 1.72 | 0.32 |
| Hematocrit [%] | 1.09 | 0.94 | 1.25 | 0.25 |
| Mean Red Blood Cells Volume [fl] | 1.01 | 0.89 | 1.15 | 0.86 |
| White Blood Cells [G/l] | 1.08 | 0.77 | 1.51 | 0.65 |
| Platelets [G/l] | 1.00 | 0.99 | 1.01 | 0.58 |
| **Mean Platelet Volume [fl]** | **2.21** | **1.07** | **4.58** | **0.03** |
| **Platelet Distribution Width [%]** | **1.54** | **1.08** | **2.20** | **0.02** |
| HbA1c [%] | 1.26 | 0.85 | 1.87 | 0.24 |
| C-reactive protein [nmol/l] | 0.88 | 0.65 | 1.19 | 0.41 |
| ALT [UI/l] | 1.08 | 0.99 | 1.17 | 0.09 |
| AST [UI/l] | 0.99 | 0.89 | 1.12 | 0.93 |
| Creatinine [µmol/l] | 1.59 | 0.03 | 80.95 | 0.82 |
| ACR [mg/mmol] | 0.99 | 0.97 | 1.02 | 0.64 |
| Triglycerides [mmol/l] | 1.00 | 0.99 | 1.01 | 0.72 |
| Total cholesterol [mmol/l] | 1.00 | 0.98 | 1.01 | 0.70 |
| LDL-C [mmol/l] | 1.00 | 0.98 | 1.02 | 0.82 |
| HDL-C [mmol/l] | 0.99 | 0.96 | 1.02 | 0.49 |
| Triglycerides / HDL-C ratio | 1.00 | 0.99 | 1.01 | 0.72 |
| Estimated Glucose Disposal Rate [mg/kg/min] | 0.92 | 0.64 | 1.32 | 0.64 |
| Skin Autofluorescence [] | 1.77 | 0.54 | 5.83 | 0.35 |
| SBP [mmHg] | 1.01 | 0.96 | 1.06 | 0.77 |
| DBP [mmHg] | 1.04 | 0.97 | 1.11 | 0.26 |
| Height [m] | 1.06 | 0.99 | 1.13 | 0.07 |
| Weight [kg] | 0.99 | 0.95 | 1.04 | 0.74 |
| Sodium [mmol/l] | 0.96 | 0.77 | 1.20 | 0.73 |
| Potassium [mmol/l] | 3.75 | 0.91 | 15.35 | 0.07 |

ACR – Albumin to Creatinine Ratio. ALT – Alanine Transaminase. AST – Aspartate Transaminase. BMI – Body Mass Index. HbA1c – Glycated Hemoglobin. HDL-C – High-Density Lipoprotein. LDL-C – Low-Density Lipoprotein. WHR – Waist-to-Hip Ratio.
